# Long non‐coding RNA SNHG6 promotes the growth and invasion of non‐small cell lung cancer by downregulating miR‐101‐3p

**DOI:** 10.1111/1759-7714.13371

**Published:** 2020-03-09

**Authors:** Ke Li, Yongxin Jiang, Xudong Xiang, Quan Gong, Chunyan Zhou, Lijuan Zhang, Qianli Ma, Li Zhuang

**Affiliations:** ^1^ Cancer Biotherapy Center, Yunnan Cancer Hospital The Third Affiliated Hospital of Kunming Medical University Kunming China; ^2^ Cancer Institute, Yunnan Cancer Hospital The Third Affiliated Hospital of Kunming Medical University Kunming China; ^3^ Choracic Surgery, Yunnan Cancer Hospital The Third Affiliated Hospital of Kunming Medical University Kunming China; ^4^ Department of Palliative Medicine, Yunnan Cancer Hospital The Third Affiliated Hospital of Kunming Medical University Kunming China

**Keywords:** Growth, invasion, miR‐101‐3p, non‐small cell lung cancer, SNHG6

## Abstract

**Background:**

The aim of this study was to determine the function of long non‐coding RNA small nucleolar RNA host gene 6 (SNHG6) in non‐small cell lung cancer (NSCLC) and its underlying mechanisms.

**Methods:**

The association of SNHG6 or miR‐101‐3p with clinicopathological characteristics and prognosis in patents with NSCLC was assessed by TCGA dataset. Cell proliferation and invasion were evaluated by MTT and Transwell assays and SNHG6‐specific binding with miR‐101‐3p was verified by bioinformatic analysis, luciferase gene report and RNA immunoprecipitation assays. qRT‐PCR and Western blot was used to assess the effects of SNHG6 on the expression of miR‐101‐3p and chromodomain Y like (CDYL) in NSCLC cells. A xenograft tumor model in vivo was established to observe the effects of SNHG6 knockdown on tumor growth.

**Results:**

We found that increased expression of SNHG6 was associated with pathological stage and lymph node infiltration, and acted as an independent prognostic factor of tumor recurrence in patients with NSCLC. Silencing SNHG6 expression repressed cell growth and invasion in vitro and in vivo, but overexpression of SNHG6 reversed these effects. Furthermore, SNHG6 was identified to act as a sponge of miR‐101‐3p, which could reduce cell proliferation and attenuate SNHG6‐induced CDYL expression. Low expression of miR‐101‐3p or high expression of CDYL was related to poor survival in patients with NSCLC.

**Conclusions:**

Our findings demonstrated that lncRNA SNHG6 contributed to the proliferation and invasion of NSCLC by downregulating miR‐101‐3p.

## Introduction

Lung cancer is one of the most common malignancies and ranks the first leading cause of cancer‐related deaths in China.^1^ Non‐small cell lung cancer (NSCLC) as a subtype of lung cancer accounts for most of its cases with high mortality worldwide.^2^ In spite of the great efforts taken to improve the curative effects of NSCLC, the prognosis of cases is poor duo to its invasiveness and metastasis.^3^ The dysregulation of coding RNAs or non‐coding RNAs is involved in the pathogenesis of NSCLC.^4^, ^5^ Therefore, identification of the promising factors may provide the early detection of NSCLC.

Small nucleolar RNA host gene 6 (SNHG6) is a long non‐coding RNA (lncRNA) which plays a critical role in cancer progression. Increasing data have indicated that the elevated expression of SNHG6 is a poor prognostic factor in esophageal squamous cell carcinoma (ESCC),^6^ facilitates the cycle progression in breast cancer^7^ and promotes cell proliferation and invasion in colorectal cancer (CRC), osteosarcoma, gastric cancer and hepatocellular carcinoma (HCC).^8^, ^9^, ^10^, ^11^ These studies suggest that SNHG6 may be a prognostic factor for cancer.

Moreover, SNHG6 acts as the sponges of microRNAs (miRNAs) to participate in the pathogenesis of multiple malignancies. Accumulating evidence shows that SNHG6 acts as an oncogenic factor by sponging miR‐4465 in ovarian clear cell carcinoma^12^ and miR‐181a‐5p/−miR‐26a/b/−214 in CRC.^13^, ^14^, ^15^ These studies indicate that SNHG6 can mediate miRNA networks to participate in cancer progression.

However, the functional role of SNHG6 in NSCLC remains undocumented. Herein, we found that increased expression of SNHG6 or decreased expression of miR‐101‐3p was associated with pathological stage and lymph node infiltration and acted as an independent prognostic factor of tumor recurrence in patients with NSCLC. Knockdown of SNHG6 expression suppressed cell proliferation and invasion in NSCLC cells in vitro and in vivo, while overexpressing SNHG6 reversed these effects. Moreover, lncRNA SNHG6 acted as a sponge of miR‐101‐3p to upregulate CDYL expression in NSCLC cells.

## Methods

### Tissue samples

The clinicopathological and prognostic data of patients with NSCLC *(n =* 362) as well as the expression levels of SNHG6, miR‐101‐3p and CDYL were downloaded from the TCGA RNA sequencing dataset (https://genome-cancer.ucsc.edu/). Ten paired lung adenocarcinoma (LAC) tissues were collected and frozen at −80°C. Informed consent was obtained by the patients and our investigations were approved by the Ethics Committee of the Third Affiliated Hospital of Kunming Medical University.

### Cell lines

NSCLC cell lines (A549, NCI‐H23, NCI‐H1993, NCI‐H522 and NCI‐H460) and BEAS‐2B used in our study were stored in our laboratory. Lentivirus‐mediated sh‐SNHG6 or negative control (sh‐NC) vectors, and virion‐packaging elements were purchased from Genechem (Shanghai, China); SNHG6 plasmids and its negative vector pcDNA3.1 as well as miR‐101‐3p mimic or inhibitor were purchased from GenePharma (Shanghai, China).

### Plasmid construction

Wild‐type (WT) SNHG6 or CDYL 3′ UTR vector containing the miR‐101‐3p binding sites, and its corresponding mutant (Mut) fragment were constructed by annealing double‐strand DNA and inserted into the pmirGLO vector at the BamHI and EcoRI sites. In addition, a shRNA targeting SNHG6 (GCGGCATGTATTGAGCATATA) was constructed by annealing single‐strand hairpin cDNA.

### Cell transfection

NSCLC cells were cultured in DMEM medium supplemented with 10% heat‐inactivated FBS, 100 U/ml of penicillin, and 100 μg/ml of streptomycin. Cells in this medium were placed in a humidified atmosphere containing 5% CO_2_ at 37°C. When cells reached 60%–80% confluence, NSCLC cells were transfected with sh‐SNHG6 or SNHG6 plasmids, and cultured at 37°C and 5% CO_2_ for four hours. The supernatant was then discarded and serum containing growth medium added.

### Quantitative RT‐PCR

To examine the expression levels of SNHG6 in NSCLC tissues and cell lines, qRT‐PCR was performed. Total RNA was extracted using TRIzol (Invitrogen, Karlsruhe, Germany) according to the manufacturer's protocol. Reverse transcription was performed by using PrimeScript RT Reagent Kit and cDNA amplification by using SYBR Premix Ex Taq (TaKaRa, Dalian, China). β‐actin or U6 gene was used as an endogenous control. The primers used are listed in Table S1.

### Western blot analysis

NSCLC cell lines were harvested and extracted using lysis buffer (Tris‐HCl, SDS, Mercaptoethanol, Glycerol). Cell extracts were boiled for five minutes in loading buffer and then equal amount of cell extracts were separated on 12% SDS‐PAGE gels. Separated protein bands were transferred into polyvinylidene fluoride (PVDF) membranes. The primary antibodies against PCNA (ab19166, abcam, Cambridge, MA, USA), MMP2 (ab97779, abcam) and CDYL (ab3999, abcam) were diluted (1:1000) according to the instructions and incubated overnight at 4°C. Further experiments were conducted as previously described.^16^

### MTT and transwell assays

MTT and Transwell assays were conducted as previously described.^16^

### Dual‐luciferase reporter assay

NSCLC cells were seeded into 24‐well plates. After 24 hours incubation, luciferase report vector carrying WT or Mut SNHG6 3′UTR was cotransfected with miR‐101‐3p mimic or inhibitor into A549 or NCI‐H460 cells. Then, 48 hours after transfection, luciferase activities were examined with a Dual‐Luciferase Reporter Assay System (Promega).

### RNA immunoprecipitation (RIP) assay

RIP assay was conducted by using a Magna RIP RNA Binding Protein Immunoprecipitation Kit (Millipore, Billerica, MA, USA) according to the manufacturer's instructions. A549 and NCI‐H460 cells were lysed by RIP buffer and cell lysis was incubated with magnetic beads coated with anti‐Ago2 or anti‐IgG antibody. The beads were incubated with Proteinase K to elute protein and RNAs from the beads. Finally, RNA was purified and SNHG6 and miR‐101‐3p levels were examined by qRT‐PCR analysis and PCR products were run on a 3% agarose gel. Fold enrichment method (2 − ^ΔΔCt^) was used to calculate the SNHG6 and miR‐101‐3p levels. RIP assays were replicated three times.

### In vivo tumorigenesis

BALB/c (nu/nu) nude mice (six weeks old, *n* = 5) were obtained from Shanghai Laboratory Animals Center (Shanghai, China). A mouse tumor model was constructed by subcutaneously injecting sh‐SNHG7 or sh‐NC stably transfected 6 × 10^7^ NCI‐H460 cells. After three weeks of monitoring the tumor size, the mice were sacrificed, and tumor tissue samples were obtained. The tumor weight and tumor size were measured every other day, and the tumor volume was calculated based on the formula: length × width^2^/2. This animal protocol was approved by the Animal Ethics Committee of the Third Affiliated Hospital of Kunming Medical University.

### Immunochemistry analysis

Immunochemistry (IHC) analysis was performed as previously reported.^16^

### Statistical analysis

SPSS 20.0 was used for statistical analysis. All values were recorded as mean ± SEM from at least three independent experiments. A two‐tailed Student's *t*‐test, analysis of variance (ANOVA) and Chi‐square test were used to evaluate the differences between both groups. Survival or recurrence curves were plotted using the Kaplan‐Meier method using a log‐rank test. Statistical significance was set at *P* < 0.05.

## Results

### Increased expression of lncRNA SNHG6 associated with poor prognosis in LAC patients

**Figure 1:**
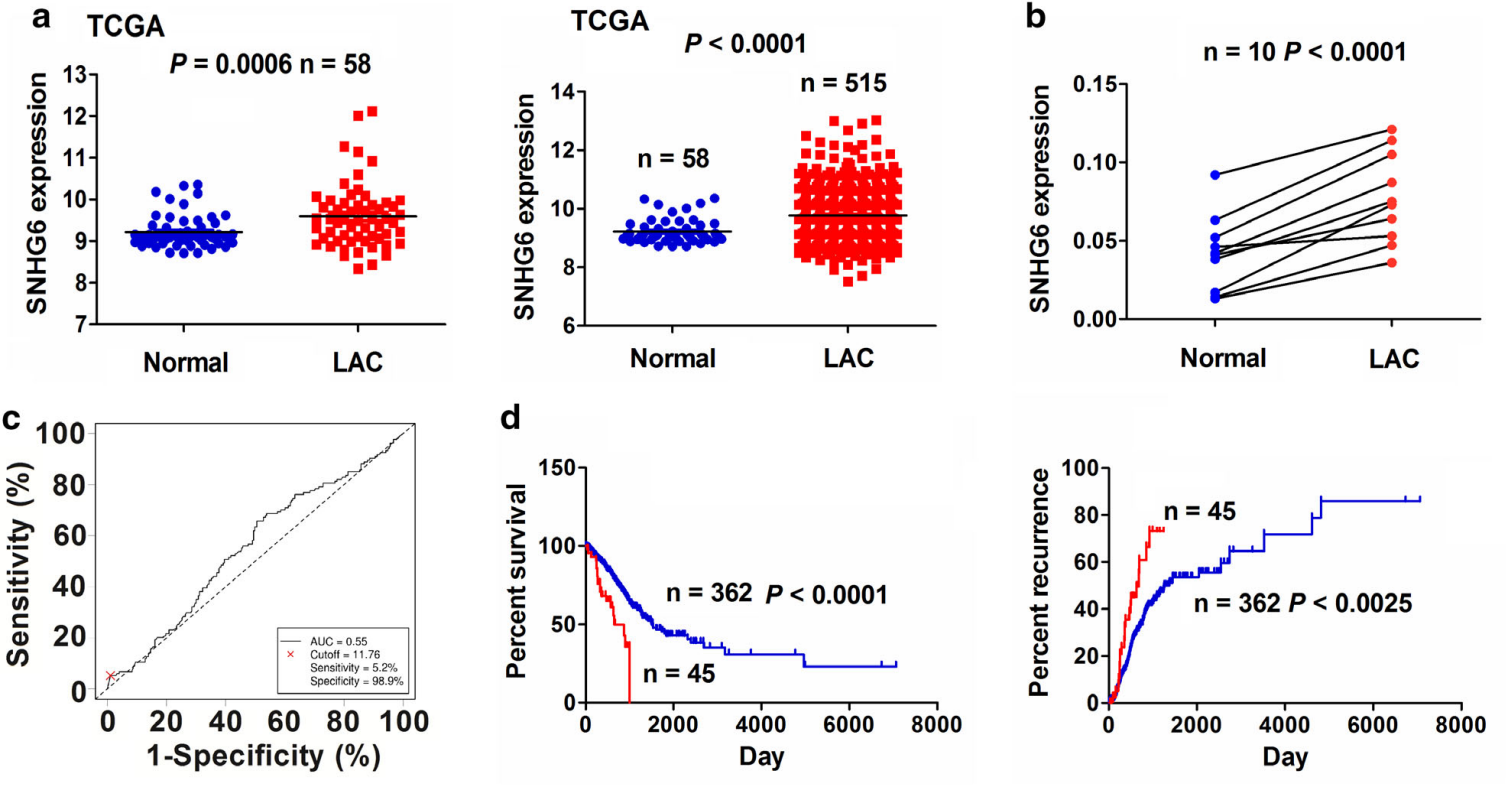
Increased expression of lncRNA SNHG6 was associated with poor survival and tumor recurrence in LAC patients. (**a**) TCGA cohort indicated an increased expression level of SNHG6 in 58 paired and 515 unpaired LAC tissues. (**b**) qRT‐PCR also showed an elevated expression level of SNHG6 in 10 paired LAC samples. (**c**) The cutoff value of SNHG6 was acquired by ROC curve in LAC according to the SNHG6 expression, and the patients' survival time and survival status by Cutoff Finder. (**d**) Kaplan‐Meier analysis demonstrated that the patients with high SNHG6 expression harbored a poorer survival and a higher tumor recurrence as compared with those with low SNHG6 expression (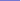
low SNHG6 expression, 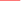 high SNHG6 expression), (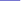
low SNHG6 expression, 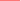 high SNHG6 expression).

We detected the expression of SNHG6 in LAC tissue samples by TCGA dataset, which indicated that SNHG6 expression levels were markedly increased in paired (*n* = 58) and unpaired LAC tissues (*n* = 515, Fig 1a). A similar result was further confirmed in 10 paired LAC tissue samples by qRT‐PCR analysis (Fig 1b). Taking into account the SNHG6 expression levels, and patients' survival time and survival status, a cutoff value (11.76) of SNHG6 was obtained in LAC using Cutoff Finder (http://molpath.charite.de/cutoff/load.jsp) (Fig 1c), and the patients were divided into high SNHG6 expression and low SNHG6 expression groups. As shown in Table 1, high expression of SNHG6 was associated with pathological stage and lymph node infiltration in LAC patients. Kaplan‐Meier analysis demonstrated that the patients with high SNHG6 expression displayed a poorer survival and a higher tumor recurrence as compared with those with low SNHG6 expression (Fig 1d).

**Table 1:** The association of SNHG6 expression with clinicopathological characteristics in LAC patients

		SNHG6		
Variables	Cases (*n*)	High	Low	*P‐*value
Total	407	45	362	
Age (years)				
≥60	293	31	262	
<60	114	14	100	0.624
Gender				
Male	184	22	162	
Female	223	23	200	0.599
Pathological stage				
I/II	327	30	297	
III/IV	80	15	65	0.014
T stage				
T1/T2	358	40	318	
T3/T4	49	5	44	0.839
N stage				
Negative	269	21	248	
Positive	138	24	114	0.004
M stage				
Negative	260	31	229	
Positive	147	14	133	0.459

LAC, lung adenocarcinoma.

Univariate Cox regression analysis indicated that high SNHG6 expression was related to an increased risk of poor survival and tumor recurrence in NSCLC (Table 2 and Table S2). Given all the confounding factors, multivariate Cox regression analysis unveiled that high expression of SNHG6 was an independent prognostic factor of tumor recurrence rather than poor survival in LAC patients (Table 2 and Table S2).

**Table 2:** Cox regression analysis of SNHG6 expression as a recurrence predictor in LAC patients

	Univariate Cox regression analysis		Multivariate Cox regression analysis	
Variables	RR (95% CI)	*P*‐value	RR (95% CI)	*P*‐value
Age (years)				
≥60 vs. <60	1.370 (0.926–2.028)	0.115	NA	NA
Gender				
Male vs. female	0.924 (0.663–1.288)	0.640	NA	NA
Pathological stage				
III/IV vs. I/II	1.619 (1.099–2.384)	0.015	1.178 (0.718–1.932)	0.516
T stage				
T3 + T4 vs. T1 + T2	1.955 (1.226–3.118)	0.005	1.841 (1.123–3.020)	0.016
N staging				
Positive vs. negative	1.490 (1.066–2.081)	0.019	1.256 (0.827–1.908)	0.286
M stage				
Positive vs. negative	0.961 (0.679–1.361)	0.824	NA	NA
SNHG6 expression				
High vs. low	2.743 (1.208–6.229)	0.016	2.428 (1.037–5.686)	0.041

LAC, lung adenocarcinoma; NA, not analyzed.

### SNHG6 promoted the proliferation and invasion of NSCLC cells

**Figure 2:**
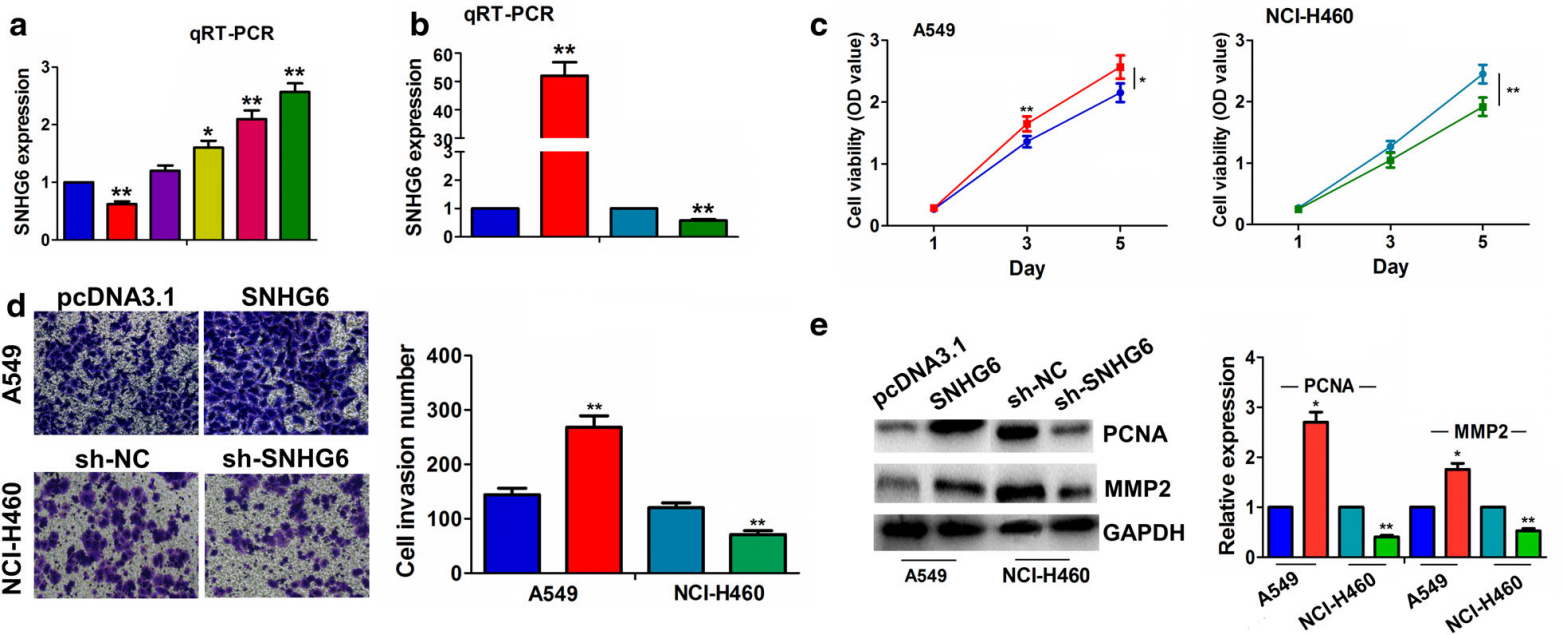
SNHG6 promoted proliferation and invasion of NSCLC cells. (**a**) qRT‐PCR analysis showed that SNHG6 had a lower expression in A549 cell lines but a higher expression in NCI‐460 cell line (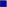
BEAS‐2B, 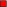 A549, 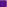 NCI‐H23, 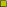 NCI‐H1993, 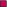 NCI‐H522, 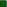 NCI‐H460). (**b**) qRT‐PCR analysis of the overexpression efficiencies of SNHG6 plasmids in A549 cell line or knockdown efficiency of sh‐SNHG6 in NCI‐H460 cell line (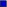
pcDNA3.1, 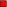 SNHG6, 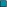 sh‐NC, 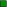 sh‐SNHG6). (**c**), (**d**), MTT and Transwell analysis of the effects of SNHG6 overexpression or knockdown on cell proliferation and invasion in A549 and NCI‐H460 cells (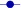
pcDNA3.1, 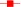 SNHG6, 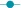 sh‐NC, 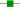 sh‐SNHG6). (**e**) Western blot analysis of the effects of SNHG6 overexpression or knockdown on PCNA and MMP2 expression in A549 or NCI‐H460 cell line (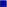
pcDNA3.1, 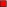 SNHG6, 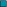 sh‐NC, 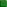 sh‐SNHG6), (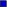 pcDNA3.1, 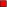 SNHG6, 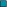 sh‐NC, 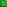 sh‐SNHG6). *P < 0.05; **P < 0.01.

We examined the expression of SNHG6 in different NSCLC cell lines and found that SNHG6 had a higher expression in NCI‐H460 cell line, but a lower expression in A549 cell line as compared with the normal cell line BEAS‐2B (Fig 2a). The overexpression efficiencies of SNHG6 plasmids in A549 cell line or the knockdown efficiency of sh‐SNHG6 in NCI‐H460 cell line were then confirmed by qRT‐PCR analysis (Fig 2b). We found that ectopic expression of SNHG6 promoted cell viability and cell invasion compared with the control group, but knockdown of SNHG6 reversed these effects (Fig 2c,d). Additionally, western blot analysis indicated that overexpressing SNHG6 upregulated PCNA and MMP2 expression in A549 cells, but silencing SNHG6, downregulated their expression in NCI‐H460 cells (Fig 2e).

### MiR‐101‐3p negatively correlated with SNHG6 expression and poor survival in LAC patients

**Figure 3:**
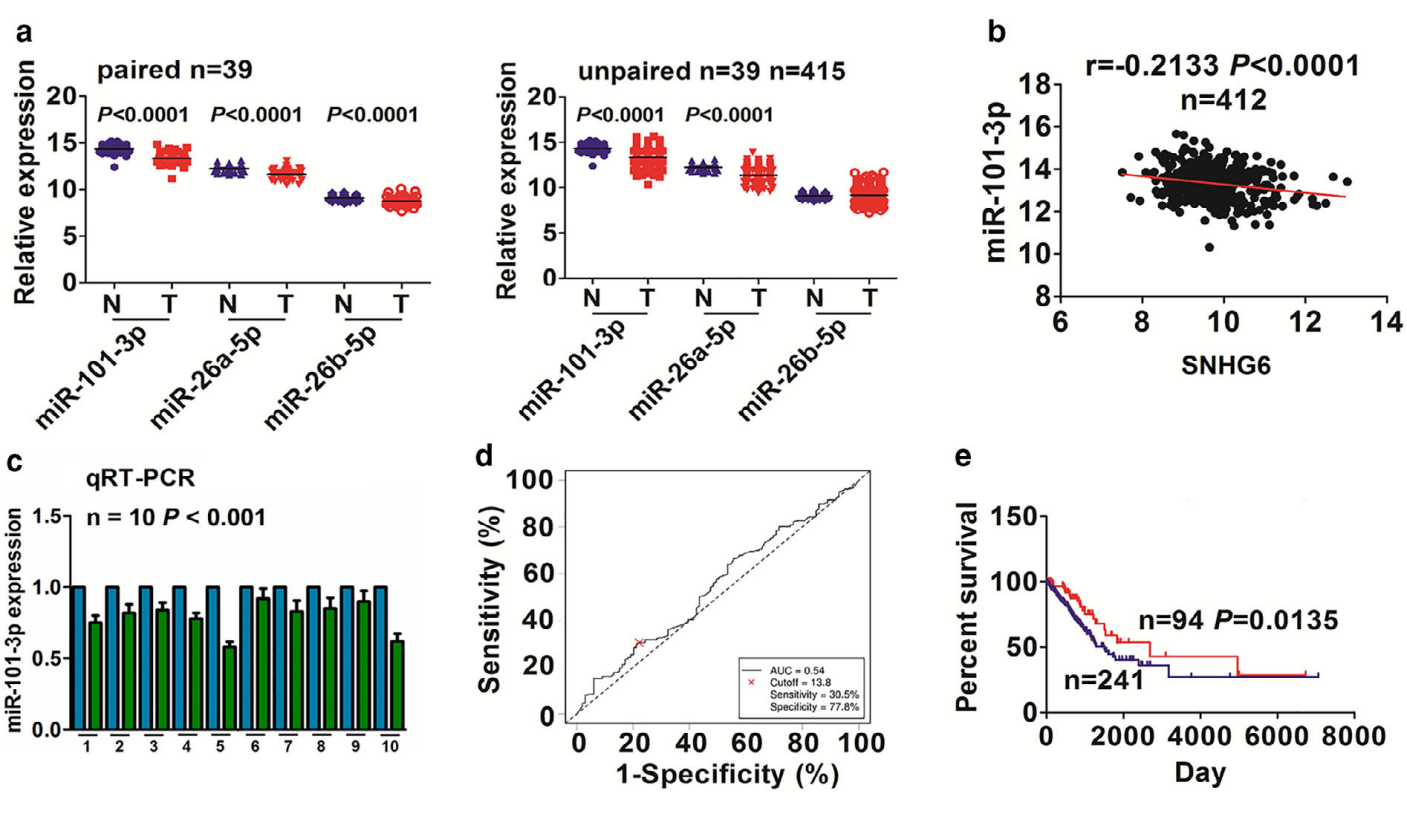
miR‐101‐3p was associated with SNHG6 expression and prognosis in LAC patients. (**a**) TCGA analysis of the expression levels of miR‐101‐3p/−26a‐5p/26b‐5p in 39 paired and 415 unpaired LAC tissues. (**b**) Pearson correlation analysis of a negative correlation of SNHG6 with miR‐101‐3p expression in LAC tissues. (**c**) qRT‐PCR indicated a decreased expression level of miR‐101‐3p in 10 paired LAC samples. (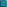) Adjacent normal and (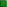) LAC. (**d**) The cutoff value of miR‐101‐3p was acquired by ROC curve in LAC according to the miR‐101‐3p expression, and the patients' survival time and survival status by Cutoff Finder. (**e**) Kaplan‐Meier analysis demonstrated that the patients with low miR‐101‐3p expression had a poorer survival as compared with those with high miR‐101‐3p expression. (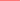) miR‐101‐3p high expression and (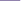) miR‐101‐3p low expression.

According to the high stringency, we used the starBasev2.0 (http://starbase.sysu.edu.cn/targetSite.php) to screen three miRNAs (miR‐101‐3p, miR‐26a‐5p and miR‐26b‐5p) that can bind with SNHG6 and found that their expression levels were decreased in 39 paired and 415 unpaired LAC tissues (Fig 3a). Pearson correlation analysis showed that miR‐101‐3p rather than miR‐26a‐5p/−26b‐5p had a negative correlation with SNHG6 expression in 412 LAC tissues (Fig 3b and Fig S1). A similar result for miR‐101‐3p expression was confirmed by qRT‐PCR analysis (Fig 3c).

We then obtained a cutoff value (13.8) of miR‐101‐3p in LAC patients (Fig 3d), and divided the patients into high miR‐101‐3p expression and low miR‐101‐3p expression groups. We found that miR‐101‐3p expression was associated with pathological stage and lymph node infiltration in NSCLC (Table S3). Kaplan‐Meier analysis demonstrated that the patients with low miR‐101‐3p expression possessed a poorer survival (Fig 3e), but had no difference in tumor recurrence (Fig S2) as compared with those with high miR‐101‐3p expression. Multivariate Cox regression analysis uncovered that miR‐101‐3p expression was not an independent prognostic factor of overall survival in patients with NSCLC (Table S4).

### SNHG6 negatively regulated miR‐101‐3p expression in NSCLC cells

**Figure 4:**
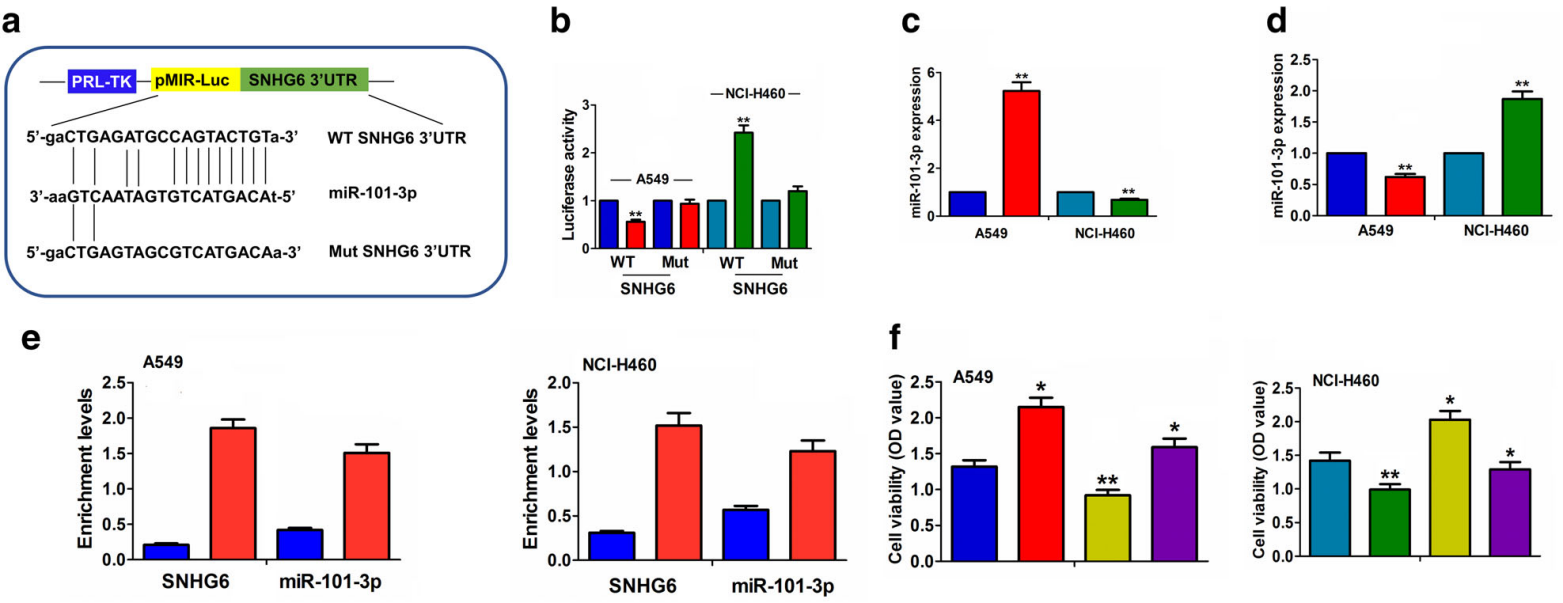
SNHG6 negatively regulated miR‐101‐3p expression in NSCLC cells. (**a**) The binding sites between miR‐101‐3p and WT or Mut SNHG6 3′UTR. (**b**) The luciferase activity of WT SNHG6 3′UTR was decreased by miR‐101‐3p mimic and increased by miR‐101‐3p inhibitor, but the luciferase activity of Mut SNHG6 3′UTR was unaffected by miR‐101‐3p inhibitor or inhibitor in A549 and NCI‐H460 cells. (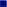) miR‐NC, (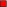) miR‐101‐3p‐mimic, (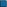) NC and (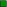) miR‐101‐3p‐inhibitor. (**c**) qRT‐PCR analysis of the overexpression efficiency of miR‐101‐3p mimic in A549 cell line or knockdown efficiency of miR‐101‐3p inhibitor in NCI‐H460 cell line. (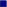) miR‐NC, (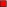) miR‐101‐3p‐mimic, (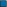) NC and (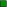) miR‐101‐3p‐inhibitor. (**d**) qRT‐PCR analysis of the effects of SNHG6 overexpression or knockdown on miR‐101‐3p expression in A549 or NCI‐H460 cell line. (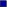) pcDNA3.1, (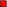) SNHG5, (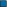) sh‐NC and (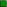) sh‐SNHG6. (**e**) RIP assay and qRT‐PCR analysis of the increased enrichment levels of SNHG6 and miR‐101‐3p pulled down from Ago2 or IgG protein in A549 and NCI‐H460 cells. (**f**) MTT analysis of the cell viability after cotransfection with SNHG6 plasmids and miR‐101‐3p mimic in A549 cell line or sh‐SNHG6 and miR‐101‐3p inhibitor in NCI‐H460 cell line.(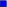) pcDNA3.1+miR‐NC, (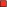) SNHG6+miR‐NC, (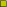) pcDNA3.1+miR‐101‐3p mimic and (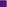) SNHG6+miR‐101‐3P mimic. (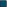) sh‐NC+NC, (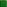) sh‐SNHG6+NC, (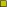) sh‐NC+miR‐101‐3p inhibitor and (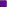) sh‐SNHG6+miR‐101‐3p inhibitor. *P < 0.05, **P < 0.01.

The binding sites between miR‐101‐3p and the WT or Mut SNHG6 3′UTR were demonstrated in Fig 4a. To further verify whether SNHG6 could bind to miR‐101‐3p, we cotransfected WT or Mut SNHG6 3′UTR and miR‐101‐3p mimic or inhibitor into A549 or NCI‐H460 cells and found that miR‐101‐3p decreased the luciferase activity of WT SNHG6 3′UTR in A549 cells, but its inhibitor reversed this effect. They had no effects on that of Mut SNHG6 3′UTR compared to the control group (Fig 4b). qRT‐PCR analysis showed that miR‐101‐3p mimic or inhibitor produced no effect on SNHG6 expression (Fig 4c and Fig S3), but overexpressing SNHG6 decreased the expression of miR‐101‐3p, and silencing SNHG6 had the opposite effect (Fig 4d). RIP assay was carried out for Ago2 protein in A549 or NCI‐H460 cells and the enrichment levels of endogenous SNHG6 and miR‐101‐3p pulled‐down from Ago2‐expressed A549 or NCI‐H460 cells were examined by qRT‐PCR analysis, indicating that, SNHG6 and miR‐101‐3p levels were markedly enriched in the Ago2 pellet as compared with those in the input control (Fig 4e). In addition, after cotransfection with SNHG6 plasmids and miR‐101‐3p mimic or sh‐SNHG6 and miR‐101‐3p inhibitor in A549 or NCI‐H460 cells, MTT assay showed that miR‐101‐3p reduced NSCLC cell viability and reversed the proliferation promoting effect of SNHG6 in A549 cell line, while miR‐101‐3p inhibitor showed the opposite effect (Fig 4f).

### CDYL identified as a direct target of miR‐101‐3p in NSCLC cells

**Figure 5:**
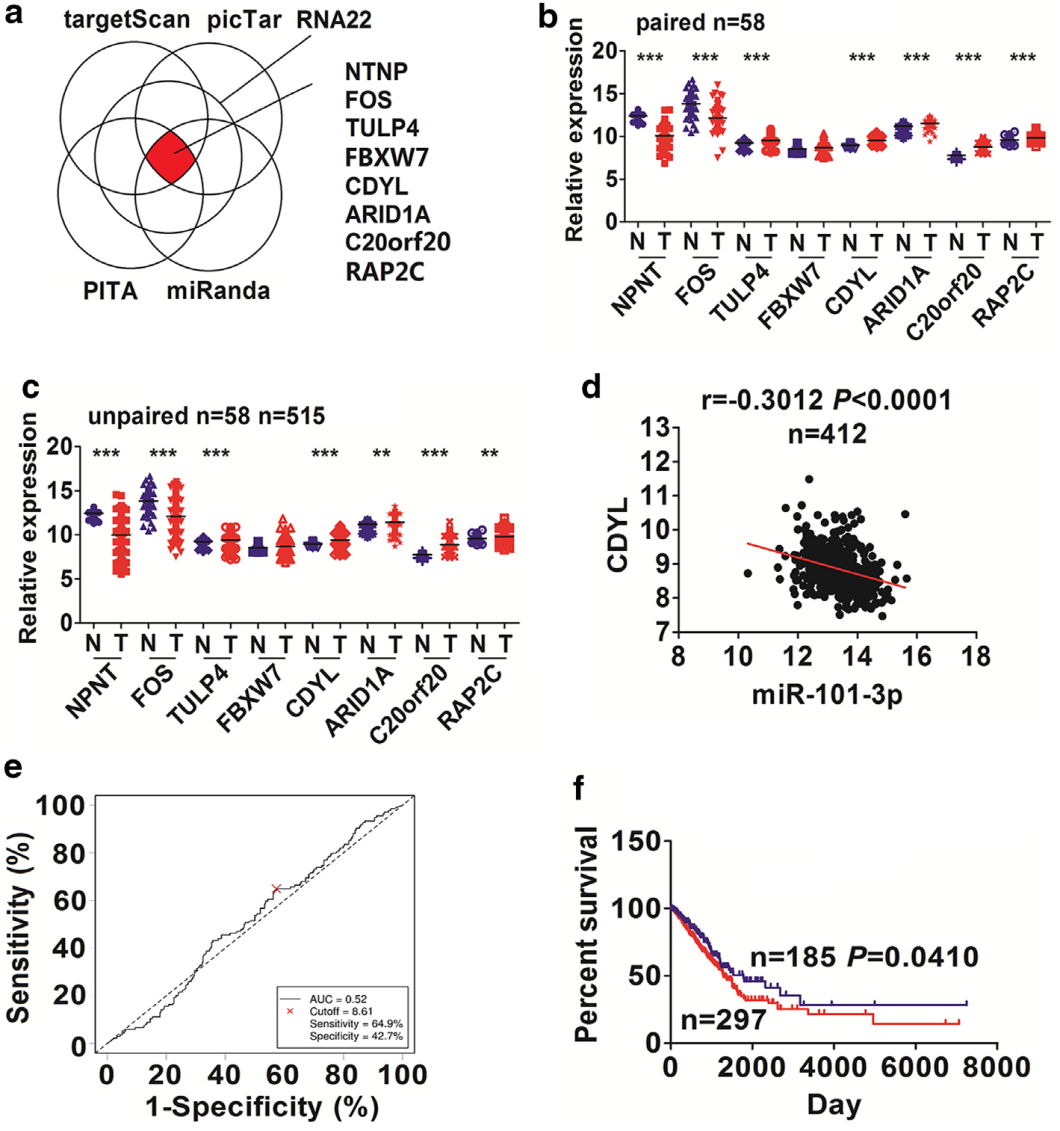
Identification of the targets of miR‐101‐3p in NSCLC tissues. (**a**) Bioinformatic identification of eight targets of miR‐101‐3p. (**b**),(**c**) TCGA analysis of the expression levels of eight targets of miR‐101‐3p in 58 paired and 515 unpaired NSCLC tissues. (**d**) Pearson analysis of the negative correlation of CDYL with miR‐101‐3p 412 expression in NSCLC tissues. (**e**) The cutoff value of CDYL was acquired by ROC curve in NSCLC according to the CDYL expression, and the patients' survival time and survival status by Cutoff Finder. (**f**) Kaplan‐Meier analysis demonstrated that the patients with high CDYL expression had a poorer survival as compared with those with low CDYL expression. (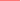) CDYL high expression and (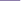) CDYL low expression.

We identified eight targets of miR‐101‐3p by using the starBasev2.0 (http://starbase.sysu.edu.cn/) (Fig 5a). The expression of these eight targets (Fig 5b,c) and their correlation with miR‐101‐3p expression in LAC tissues were subsequently analyzed, indicating that only CDYL had a most obvious negative correlation with miR‐101‐3p expression in LAC tissues (Fig 5d and Fig S4).

We then obtained a cutoff value (8.61) of CDYL in LAC (Fig 5e), and divided the patients into high CDYL expression and low CDYL expression groups. We found that CDYL expression was associated with age in NSCLC (Table S5). Kaplan‐Meier analysis indicated that the patients with high CDYL expression had a poorer survival (Fig 5f), but there was no difference in tumor recurrence (Fig S5) as compared with those with low CDYL expression. Multivariate Cox regression analysis showed that high expression of CDYL was not an independent prognostic factor of poor survival in patients with NSCLC (Table S6).

### MiR‐101‐3p reversed SNHG6‐induced CDYL expression in NSCLC cells

**Figure 6:**
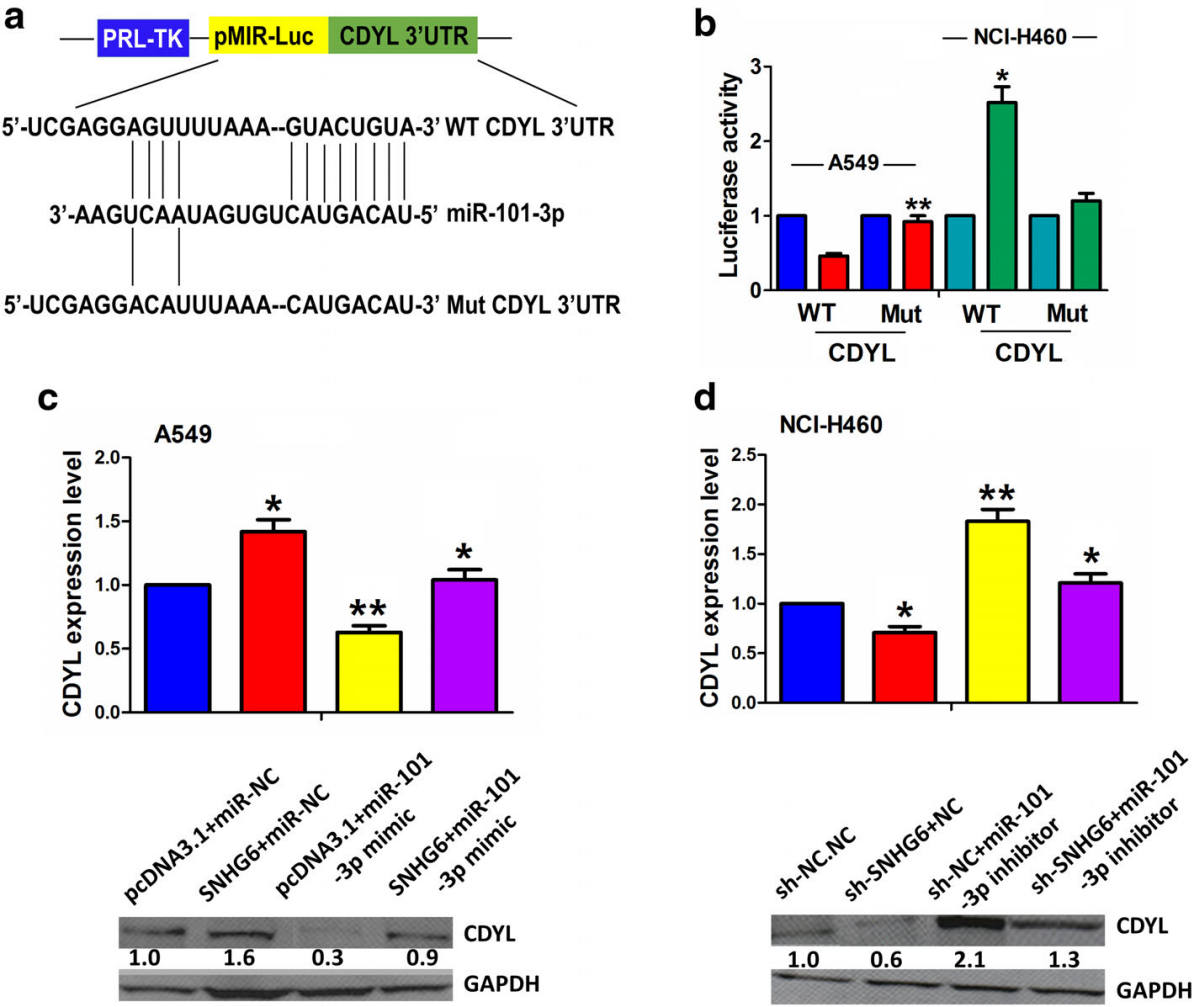
miR‐101‐3p reversed SNHG6‐induced CDYL expression in NSCLC cells. (**a**) The binding sites between miR‐101‐3p and WT or Mut CDYL 3′ UTR. (**b**) The luciferase activity of WT CDYL 3′UTR was decreased by miR‐101‐3p mimic and increased by miR‐101‐3p inhibitor, but the luciferase activity of Mut CDYL 3′UTR was unaffected by miR‐101‐3p inhibitor or inhibitor in A549 and NCI‐H460 cells. (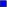) miR‐NC, (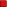) miR‐101‐3p‐mimic, (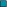) NC and (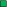) miR‐101‐3p‐inhibitor. (**c**),(**d**) qRT‐PCR and western blot analysis of the CDYL expression levels after cotransfection with SNHG6 plasmids and miR‐101‐3p mimic in A549 cell line or sh‐SNHG6 and miR‐101‐3p inhibitor in NCI‐H460 cell line. (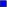) pcDNA3.1+miR‐NC, (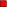) SNHG6+miR‐NC, (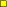) pcDNA3.1+miR‐101‐3p mimic and (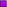) SNHG6+miR‐101‐3P mimic. (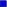) sh‐NC+NC, (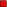) sh‐SNHG6+NC, (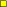) sh‐NC+miR‐101‐3p inhibitor and (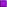) sh‐SNHG6+miR‐101‐3p inhibitor. *P < 0.05; **P < 0.01.

The binding sites between miR‐101‐3p and WT or Mut CDYL 3′UTR are demonstrated in Fig 6a. To confirm whether miR‐101‐3p could bind to CDYL 3′UTR, we cotransfected WT or Mut CDYL 3′UTR and miR‐101‐3p mimic or inhibitor into A549 or NCI‐H460 cells and found that miR‐101‐3p decreased the luciferase activity of WT CDYL 3′UTR in A549 cells, but its inhibitor reversed this effect. They had no effects on that of Mut CDYL 3′UTR compared to the control group (Fig 6b). In addition, after cotransfection with SNHG6 plasmids and miR‐101‐3p mimic in A549 cells or sh‐SNHG6 and miR‐101‐3p inhibitor in NCI‐H460 cells, qRT‐PCR and western blot analysis showed that miR‐101‐3p downregulated CDYL expression and reversed SNHG6‐induced CDYL expression in A549 cells (Fig 6c), while miR‐101‐3p inhibitor showed the opposite effect (Fig 6d).

### Knockdown of SNHG6 inhibited tumor growth in vivo

**Figure 7:**
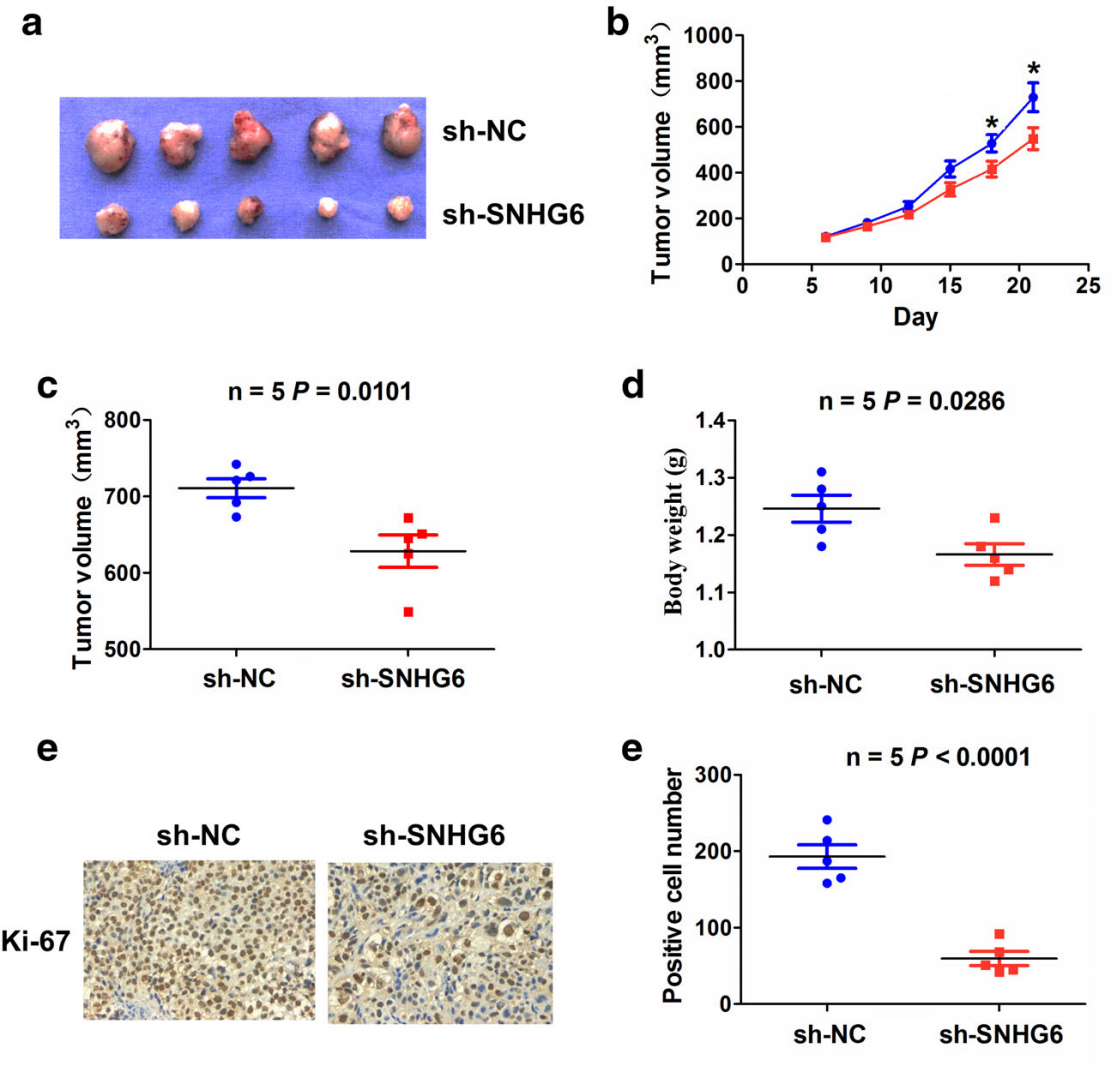
SNHG6 knockdown inhibited tumor growth in vivo. (**a**) Schematic representation of the comparison of subcutaneous xenograft tumors between sh‐NC and sh‐SNHG6 groups. (**b**) Tumor size was examined every other day and the tumor growth curve was drawn in sh‐SNHG6 and sh‐NC groups (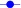
sh‐NC and 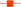
sh‐SNHG6). (**c**),(**d**), Tumor volume and weight were lower in the sh‐SNHG6 group as compared with the sh‐NC group. (**e**), IHC analysis demonstrated that Ki‐67 proliferation index was decreased in the sh‐SNHG6 group as compared with the sh‐NC group. *P < 0.05.

The effects of SNHG6 on cell growth in vivo were further investigated. As shown in Fig 7a,b and Fig S6, the tumor growth was markedly restrained in sh‐SNHG6 transfected NCI‐H460 cells in mice as compared with the sh‐NC group. After three weeks, the mice were sacrificed and the tumor tissues were harvested. We found that tumor volume and weight were lower in the sh‐SNHG6 group than that in the sh‐NC group (Fig 7c,d). In addition, IHC analysis indicated that Ki‐67 index was reduced in the sh‐SNHG6 group as compared with the sh‐NC group (Fig 7e).

## Discussion

Multiple studies have indicated that upregulation of SNHG6 is associated with poor prognosis, lymph node metastasis and TNM stage in patients with ESCC,^6^ CRC^8^ and HCC,^11^ but its expression has been shown to be downregulated in CRC.^17^ SNHG6 is also upregulated in lung adenocarcinoma (LUAD) and positively associated with TNM stage and tumor size in LUAD patients.^18^ However, the prognostic significance of SNHG6 in patients with NSCLC is unclear. Consistent with a previous study,^17^ we found that SNHG6 expression was increased and associated with poor prognosis, pathological stage and lymph node infiltration, acting as an independent prognostic factor of tumor recurrence in patients with NSCLC.

Previous studies have showed that SNHG6 can act as an oncogenic factor^6^, ^7^, ^8^, ^9^ or a tumor suppressor in cancers.^17^ On the one hand, SNHG6 facilitates tumor cell progression by activating JNK or TGF‐β/Smad signaling,^8,10^ and on the other it suppresses cell proliferation and metastasis by inactivation of the PI3K/AKT/mTOR signaling in CRC.^17^ In the present study, consistent with a previous report,^18^ we found that knockdown of SNHG6 suppressed cell proliferation and invasion in vitro and in vivo, but overexpressing SNHG6 reversed these effects. Our studies suggested that SNHG6 might act as a tumor promoting factor in NSCLC.

It is known that the regulatory mechanisms of SNHG6 are mediated by sponging a variety of miRNAs.^12^, ^13^, ^14^, ^15^ SNHG6 also acts as a competing endogenous RNA to upregulate E2F7 by sponging miR‐26a‐5p in LUAD.^18^ Likewise, we found that SNHG6 could bind with Ago2/miR‐101‐3p complex and negatively regulate miR‐101‐3p expression, indicating that SNHG6 might act as a sponge of miR‐101‐3p to promote NSCLC cell growth. In addition, lncRNA PTAR, SPRY4‐IT1 and LINC01303 also sponge miR‐101‐3p to prompt cell proliferation and metastasis in GC and ovarian cancer.^19^, ^20^, ^21^

Some studies have demonstrated that miR‐101‐3p acts as a tumor suppressor in multiple cancers. It can inhibit cell growth and metastasis and facilitate chemotherapeutic sensitivity by targeting Pim‐1, TRIM44 or EZH2.^22^, ^23^, ^24^ Moreover, miR‐101‐3p suppresses the growth and metastasis of NSCLC by inactivation of PI3K/AKT signaling.^25^ Coinciding with this study, we found that miR‐101‐3p was downregulated in NSCLC tissues and its low expression was associated with poor survival in patients with NSCLC. Chromodomain Y‐like (CDYL) was reported to indicate a poor prognosis and enhance the chemoresistance in small cell lung cancer.^26^ We herein found that upregulation of CDYL was associated with poor survival in NSCLC and acted as a direct target of miR‐101‐3p in NSCLC cells. MiR‐101‐3p reversed SNHG6 induced cell proliferation and CDYL expression. Our findings indicated that SNHG6 might act as a sponge of miR‐101‐3p to upregulate CDYL expression, thereby contributing to NSCLC.

In summary, our findings demonstrated that upregulation of SNHG6 expression was associated with pathological stage and lymph node infiltration and acted as an independent prognostic factor of tumor recurrence in LAC patients. SNHG6 promoted cell proliferation and invasion of NSCLC cells by sponging miR‐101‐3p.

## Disclosure

The authors declare that they have no conflicts of interest.

## Supporting information


**Appendix S1** Supporting information.
