# Supplementary material for: Long non‐coding RNA SNHG6 promotes the growth and invasion of non‐small cell lung cancer by downregulating miR‐101‐3p
Source: Thorac Cancer. 2020 Mar 9;11(5):1180–90. doi: 10.1111/1759-7714.13371 (PMC7180593; doi:10.1111/1759-7714.13371)
Supplement: Supplementary file 1 — Appendix S1 Supporting information. [file TCA-11-1180-s001.docx]

**Supplementary Figure legends**

**Supplementary Figure S1.** Pearson analysis of the correlation of SNHG6 with miR-26a-5p/-26b-5p expression in NSCLC tissues.

**Supplementary Figure S2.** Kaplan-Meier analysis of the association of high or low miR-101-3p expression with tumor recurrence in patients with NSCLC.

**Supplementary Figure S3.** qRT-PCR analysis of the effects of miR-101-3p mimic or inhibitor on SNHG6 expression in A549 or NCI-H460 cell line.

**Supplementary Figure S4.** Pearson analysis of the correlation of miR-101-3p with the expression of C20orf20, RAP2C, TULP4 and ARID1A in NSCLC tissues.

**Supplementary Figure S5.** Kaplan-Meier analysis of the association of high or low CDYL expression with tumor recurrence in patients with NSCLC.

**Supplementary Figure S6.** Schematic representation of the comparison of subcutaneous xenograft tumors between sh-SNHG6 and control sh-NC groups,

**Supplementary Tables**

**Table S1** List of primers

| Gene | Sense (F) | Anti-sense (R) |
| --- | --- | --- |
| SNHG6 | 5’ GCGAGGTGCAAGAAAGCC3’ | 5’ :ACATGCCGCGTGATCCTA3’ |
| GAPDH | 5’ CCTCTTGGCCGTTTTTCTCCA3’ | 5’ CCTCTTGGCCGTTTTTCTCCA3’ |
| miR-101-3p | 5’ACGGGCGAGCTCAGTACTGTG3’ | 5’CCAGTGCAGGGTCCGAGCTA 3’ |
| U6 | 5’CCCTGGCACCCAGCAC3’ | 5’GCCGATCCACACGGAGTAC3’ |
| CDYL | 5’ CTTATCCGATGGAAAGGCTACG3’ | 5’ GCTTCCCTGACTTGATCCTCTT 3’ |

**Table S2** Cox regression analysis of SNHG6 expression as a survival predictor in LAC patients

| Variables | Univariate Cox regression analysis | |  | Multivariate Cox regression analysis | |
| --- | --- | --- | --- | --- | --- |
|  | RR (95% CI) | *P* value |  | RR (95% CI) | *P* value |
| *Age (years)* |  |  |  |  |  |
| ≥60 vs. <60 | 1.006 (0.691 to 1.466) | 0.974 |  | NA | NA |
| *Gender* |  |  |  |  |  |
| Male vs. Female | 1.042 (0.741 to 1.464) | 0.814 |  | NA | NA |
| *Pathological stage* |  |  |  |  |  |
| Ⅲ/Ⅳ vs.Ⅰ/Ⅱ | 2.399 (1.669 to 3.447) | <0.0001 |  | 1.462 (0.915 to 2.335) | 0.112 |
| *T stage* |  |  |  |  |  |
| T3+T4 vs. T1+T2 | 2.171 (1.378 to 3.420) | 0.001 |  | 1.768 (1.088 to 2.872) | 0.021 |
| *N staging* |  |  |  |  |  |
| Positive vs. Negative | 2.236 (1.592 to 3.139) | <0.0001 |  | 1.697 (1.111 to 2.593) | 0.014 |
| *M stage* |  |  |  |  |  |
| Positive vs. Negative | 0.969 (0.675 to 1.392) | 0.866 |  | NA | NA |
| *SNHG6 expression* |  |  |  |  |  |
| High VS. Low | 2.965 (1.380 to 6.730) | 0.005 |  | 2.067 (0.940 to 4.547) | 0.071 |

NA: not analyzed

**Table S3** The association of miR-101-3p expression with clinicopathological

characteristics in patients with NSCLC

| Variables | Cases  (n) | miR-101-3p | | *P* value |
| --- | --- | --- | --- | --- |
|  |  | High | Low |  |
| Total | 335 | 94 | 241 |  |
| *Age (years)* |  |  |  |  |
| ≥60 | 240 | 69 | 171 |  |
| <60 | 95 | 25 | 70 | 0.688 |
| *Gender* |  |  |  |  |
| Male | 154 | 42 | 112 |  |
| Female | 181 | 52 | 129 | 0.768 |
| *Pathological stage* |  |  |  |  |
| Ⅰ/Ⅱ | 274 | 84 | 190 |  |
| Ⅲ/Ⅳ | 61 | 10 | 51 | 0.025 |
| *T stage* |  |  |  |  |
| T1/T2 | 292 | 85 | 207 |  |
| T3/T4 | 43 | 9 | 34 | 0.363 |
| *N stage* |  |  |  |  |
| Negative | 229 | 73 | 156 |  |
| Positive | 106 | 21 | 85 | 0.022 |
| *M stage* |  |  |  |  |
| Negative | 202 | 50 | 152 |  |
| Positive | 133 | 44 | 89 | 0.107 |

**Table S4** Cox regression analysis of miR-101-3p as a survival predictor in LAC patients

| Variables | Univariate Cox regression analysis | |  | Multivariate Cox regression analysis | |
| --- | --- | --- | --- | --- | --- |
|  | RR (95% CI) | *P* value |  | RR (95% CI) | *P* value |
| *Age (years)* |  |  |  |  |  |
| <60 vs. ≥60 | 1.005 (0.648 to 1.559) | 0.983 |  | NA | NA |
| *Gender* |  |  |  |  |  |
| Male vs. Female | 1.121 (0.755 to 1.664) | 0.572 |  | NA | NA |
| *Pathological stage* |  |  |  |  |  |
| Ⅲ/Ⅳ vs.Ⅰ/Ⅱ | 2.327 (1.518 to 3.567) | <0.0001 |  | 1.296 (0.735 to 2.284) | 0.370 |
| *T stage* |  |  |  |  |  |
| T3+T4 vs. T1+T2 | 2.247 (1.354 to 3.729) | 0.002 |  | 1.839 (1.071 to 3.158) | 0.027 |
| *N staging* |  |  |  |  |  |
| Positive vs. Negative | 2.262 (1.524 to 3.359) | <0.0001 |  | 1.772 (1.073 to 2.928) | 0.025 |
| *M stage* |  |  |  |  |  |
| Positive vs. Negative | 0.945 (0.628 to 1.423) | 0.788 |  | NA | NA |
| *miR-101-3p expression* |  |  |  |  |  |
| High VS. Low | 0.553 (0.343 to 0.891) | 0.015 |  | 0.618 (0.417 to 1.112) | 0.124 |

NA: not analyzed

**Table S5** The association of CDYL expression with clinicopathologic

characteristics of LAC patients

| Variables | Cases  (n) | CDYL | | *P* value |
| --- | --- | --- | --- | --- |
|  |  | High | Low |  |
| Total | 407 | 244 | 163 |  |
| *Age (years)* |  |  |  |  |
| ≥60 | 293 | 165 | 128 |  |
| <60 | 114 | 79 | 35 | 0.018 |
| *Gender* |  |  |  |  |
| Male | 184 | 119 | 65 |  |
| Female | 223 | 125 | 98 | 0.085 |
| *Pathological stage* |  |  |  |  |
| Ⅰ/Ⅱ | 327 | 198 | 129 |  |
| Ⅲ/Ⅳ | 80 | 46 | 34 | 0.614 |
| *T stage* |  |  |  |  |
| T1/T2 | 358 | 215 | 143 |  |
| T3/T4 | 49 | 29 | 20 | 1.000 |
| *N stage* |  |  |  |  |
| Negative | 269 | 155 | 114 |  |
| Positive | 138 | 89 | 49 | 0.200 |
| *M stage* |  |  |  |  |
| Negative | 260 | 162 | 98 |  |
| Positive | 147 | 82 | 65 | 0.208 |

**Table S6** Cox regression analysis of CDYL expression as survival predictor

| Variables | Univariate Cox regression analysis | |  | Multivariate Cox regression analysis | |
| --- | --- | --- | --- | --- | --- |
|  | RR (95% CI) | *P* value |  | RR (95% CI) | *P* value |
| *Age (years)* |  |  |  |  |  |
| <60 vs. ≥60 | 1.006 (0.691 to 1.466) | 0.974 |  | NA | NA |
| *Gender* |  |  |  |  |  |
| Male vs. Female | 1.042 (0.741 to 1.464) | 0.814 |  | NA | NA |
| *Pathological stage* |  |  |  |  |  |
| Ⅲ/Ⅳ vs.Ⅰ/Ⅱ | 2.399 (1.669 to 3.447) | <0.0001 |  | 1.474 (0.921 to 2.360) | 0.106 |
| *T stage* |  |  |  |  |  |
| T3+T4 vs. T1+T2 | 2.171 (1.378 to 3.420) | 0.001 |  | 1.725 (1.063 to 2.799) | 0.027 |
| *N staging* |  |  |  |  |  |
| Positive vs. Negative | 2.236 (1.592 to 3.139) | <0.0001 |  | 1.740 (1.141 to 2.654) | 0.010 |
| *M stage* |  |  |  |  |  |
| Positive vs. Negative | 0.969 (0.675 to 1.392) | 0.866 |  | NA | NA |
| *C20orf20 expression* |  |  |  |  |  |
| High VS. Low | 1.269 (0.909 to 1.849) | 0.152 |  | 1.185 (0.827 to 1.698) | 0.355 |

NA: not analyzed
